# Poison by Tartaric Acid

**Published:** 1845-12

**Authors:** 


*Poison by Tartaric Acid.*—It has been questioned if this acid be a poison. Pommer and M. Orfila are for the affirmative; Coindet and Christison for the negative. The following fact strengthens the opinion of the two first named: William Wats, being affected with rheumatism, applied the 7th Dec., 1844, to Charles Watkins, druggist, to purchase 2 ℥ of Epsom salts. Before leaving, the thought suddenly occurred to him of changing it for another salt less bitter. This was granted to him, and having returned home and dissolved the new article given to him, he swallowed it. His face, some moments after this, became red. He cried out he was poisoned, and then ceased to speak. Other symptoms were developed, and Mr. Wats died on the 16th. Mr. Brood, charged with the examination of what remained in the glass from which he had drank, recognized Tartaric Acid. The apothecary, Mr. Watkins, confessed his error, and attributed it to the change which some one had made of the bottle of the acid, for that commonly occupied by an insipid salt.—*Pharmaceutical Journal.—Southern Medical Journal.*

